# Case Report: Primary osteosarcoma of the kidney

**DOI:** 10.3389/fonc.2023.1175518

**Published:** 2023-10-09

**Authors:** Junyong Chen, Hongxian Liao, Rungen Zhan, Qiaoli Zheng, Jian Deng, Guojie Wang, Jie Zhang

**Affiliations:** ^1^Department of Urology, Zhuhai People’s Hospital (Zhuhai hospital affiliated to Jinan University), Zhuhai, China; ^2^School of the first clinical medicine, Guangdong Medical University, Zhanjiang, China; ^3^Department of Radiology, Zhuhai People’s Hospital (Zhuhai hospital affiliated to Jinan University), Zhuhai, China; ^4^Department of Pathology, Zhuhai People’s Hospital (Zhuhai hospital affiliated to Jinan University), Zhuhai, China; ^5^Department of Radiology, Fifth Affiliated Hospital of Sun Yat-sen University, Zhuhai, China; ^6^Department of Radiology, The First People’s Hospital of Kashi Prefecture, Xinjiang Uygur Autonomous Region, China

**Keywords:** osteosarcoma, extraosseous, kidney, MRI, CT

## Abstract

Extraosseous osteosarcoma is a rare malignant tumor, most commonly occurring in the thigh, upper limbs, and retroperitoneum. However, there are only a few reported cases of renal osteosarcoma. Herein, we present the case of a 54-year-old woman with malignant extraosseous osteosarcoma of the left kidney. CT and MR imaging revealed a soft tissue mass originating from the left kidney.

## Introduction

Primary renal osteosarcoma is an uncommon extraosseous malignant tumor that arises in the kidneys. While there have been sporadic cases reported in the literature, there’s still limited understanding of the variations in clinical presentations and prognoses. Our report provides a unique case with specific imaging and genetic findings, adding to the existing pool of knowledge. Primary renal osteosarcoma mostly affects adults and has a poor overall prognosis (1). Nonspecific features and intra-abdominal location make detection difficult until late into disease progression (2). Here, we present a case of primary osteosarcoma of the kidney and review its clinical features, diagnosis, and treatment options.

## Case report

**Figure 1:**
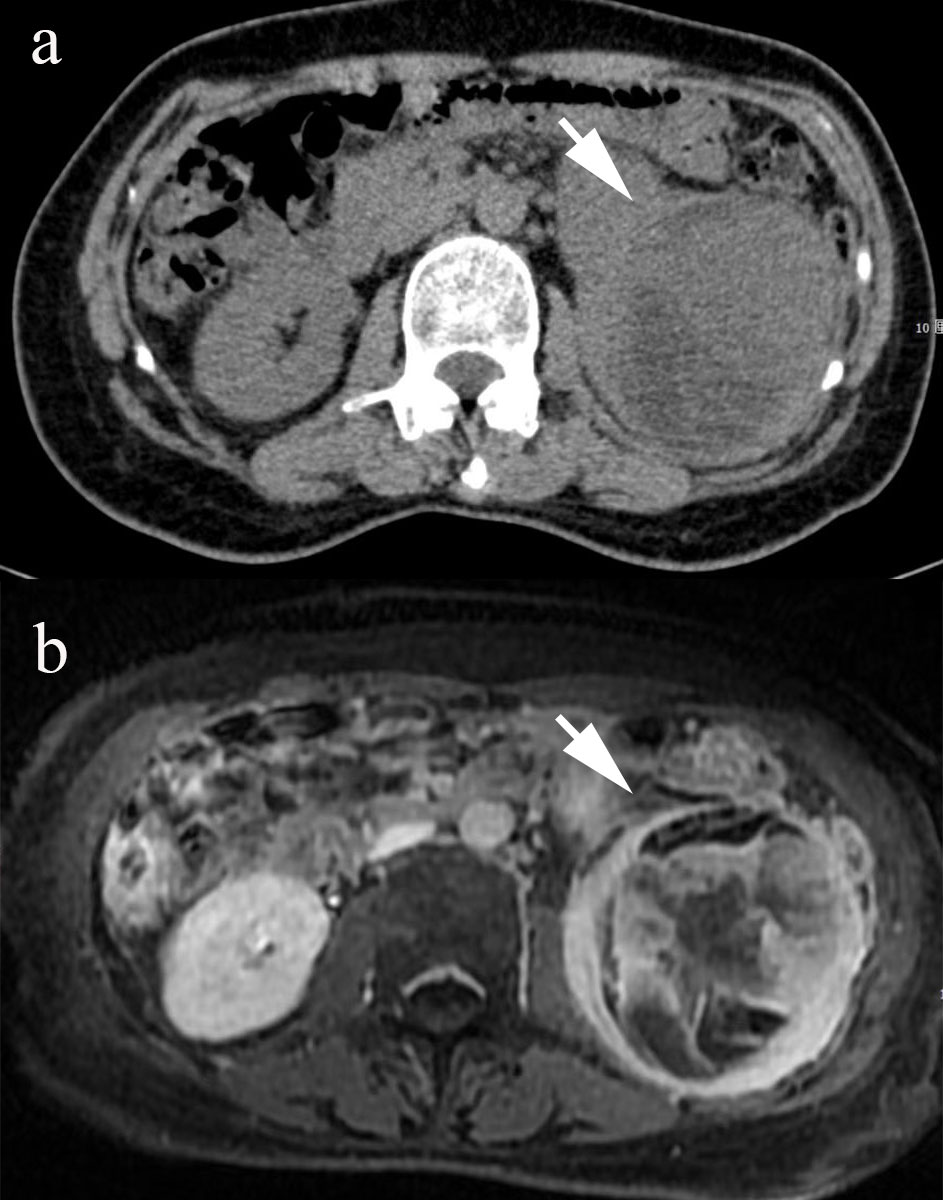
The CT image shows a mixed-density mass in the left kidney (**(A)**, arrow). The MR examination shows a mixed solid-cystic mass with unclear boundaries in the left kidney (**(B)**, arrow).

A 54-year-old female farmer presented with a 6-month history of abdominal pain, and 4 months of weight loss of approximately 5 kg. She had left abdominal pain with no tenderness, accompanied by a mass for 6 months. Physical examination revealed no tenderness in the abdomen. Abdominal computed tomography (CT) (**Figure 1A**) revealed a mixed-density mass in the left kidney. The mass displayed heterogeneous attenuation with clear boundaries, measuring approximately 8 × 6 cm in size. Magnetic resonance imaging (MRI) revealed a mixed solid cystic mass in the left kidney (**Figure 1B**). The mass had unclear boundaries, possibly indicating local invasion. A routine urine examination revealed an NWBC count of 5 U/L. All other biochemical profiles were within normal limits. The patient has no significant family history of any malignancies or known genetic predispositions. Her occupation as a farmer did not expose her to any known risk factors associated with renal osteosarcoma. We surgically excised the lesion, which measured approximately 8 × 6 × 5 cm. There were no enlarged retroperitoneal lymph nodes. Postoperative pathology revealed a left renal primary osteosarcoma. No recurrence was observed 1 year after surgery.

**Figure 2:**
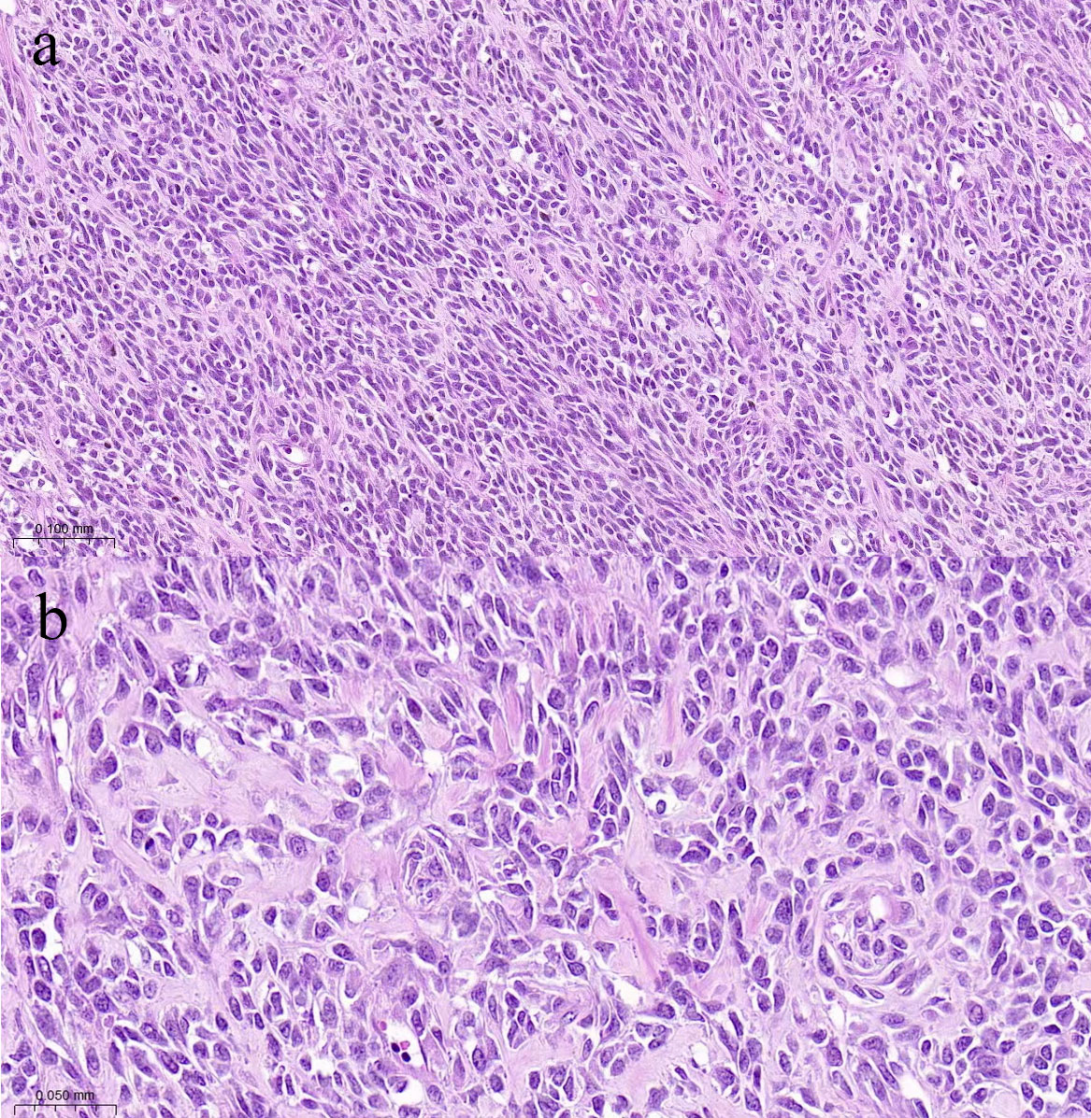
Photomicrograph shows many densely distributed invasive tumor cells are arranged in bundles and braided shapes, with collagen-rich interstitial fluid (**(A)**, HE×100). The size of tumor cells varies, while the cytoplasm and chromatin are coarse, and partial mitosis is present (**(B)**, HE×200).

Pathological findings: After gross examination, the kidney revealed an 8 × 6 cm tumor replacing the lower part of the left kidney. The surface cut showed cystic and solid masses with hemorrhagic areas. Microscopic examination using hematoxylin–eosin staining revealed numerous spindle cell proliferations with interspersed osteoid calcifications (**Figure 2**). Immunohistochemistry: vimentin+, CD4+, CD34, CD99, CD117, desmin, myogenin, S-100, EMA, Fly-1, and Dog-1 were negative, and the Ki-67 positivity rate was approximately 40%. Therefore, the patient was diagnosed with osteosarcoma. Genetic tests identified three variants (PIK3CA, CTCF, and RASA1) among the 733 genes tested; The patient had an uneventful postoperative recovery. Regular follow-up was conducted to monitor the patient’s condition, which included CT scans and MR scans. This comprehensive follow-up schedule allowed for close observation of any potential recurrence or complications. No recurrence was observed after 13 months of diligent follow-up. The patient provided informed consent for the publication of her clinical and radiological data.

## Discussion

Extraosseous osteosarcoma (EOS) is a rare malignant subtype of osteosarcoma that accounts for about 1% of all soft tissue sarcomas (1) and shares histological features with primary bone osteosarcoma. EOS occurs in all age groups and the male-to-female ratio is 1.9:1 (2). The pathological subtypes of extraosseous osteosarcoma can be divided into telangiectasis, chondroblasts, fibroblasts, osteoblasts, and small cell types. Flank pain and hematuria are the most common complaints in renal osteosarcoma (3).

The exact etiology of EOS remains unclear. According to Virchow’s theory (4), the risk factors include exposure to X-rays and radioactive substances. Under certain circumstances, such as radiation, metaplastic transformation occurs from the connective tissue to the embryonic mesenchyme, which can differentiate into osteoblasts and bones.

Common symptoms of renal osteosarcoma are flank pain and hematuria, with weight loss and a progressively enlarged soft tissue mass in the hypochondrium. Some patients experience an occult onset and pain. In most cases, by the time patients go to a clinic, the mass would have grown quite large (5). Hence, patients often have a concealed symptom onset.

Historically, EOS in kidneys has been an elusive diagnosis with only 28 cases documented, including this case reported here. The manifestation in our patient, particularly the imaging and genetic findings, aligns yet somewhat differs from previous reports, emphasizing the heterogeneity of this disease. Flank pain and hematuria, as observed in our patient, are the most common complaints in renal osteosarcoma (6). However, a comprehensive review of the existing literature, including a notable study in the American Journal of Clinical Pathology (6), showcased a range of clinical presentations and outcomes. Such variations underscore the need for a consolidated case table, which we provide below, contrasting our findings against the historical cases (**Table 1**) (3, 7–26).

**Table 1:** Summary of clinical and pathologic features of 29 primary renal osteosarcoma cases.

Characteristic	Value
Age range (mean; median), y Sex Male Female Initial symptoms Weight loss Palpable mass Flank pain, weight loss Flank pain, palpable mass Pelvic and back pain Back pain Flank pain Flank pain, gross hematuria Histotype Classic (NOS) Pleomorphic Osteoblastic Chondroblastic Low grade TNM stage pT1a N0M0 pT1b N0M0 pT2aN0M0 pT3a N0M1 pT3aN1M1 pT4N0M1 pT4N1M1 Treatment RN RN + ChT RN + RT Side Left Right Follow-up, mo DOD (range, 2 wk to 32 mo; mean, 15 mo) AWD (both at 6-mo follow-up) NED (range, 13-72 mo; mean, 27 mo) DOC (range, 9-10 mo; mean, 9.5 mo)	47-81 (59; 59) 17 (59) 12 (37) 5 (17) 8 (28) 7 (24) 2 (7) 1 (3) 1 (3) 3 (10) 2 (7) 12 (41) 11 (38) 3 (10) 2 (7) 1 (3) 1 (3) 1 (3) 1 (3) 9 (27) 1 (3) 1 (3) 15 (52) 15 (52) 10 (34) 9 (31) 18 (62) 11 (39) 21 (76) 3 (10) 4 (7) 2 (7)

AWD, alive with disease indicates metastases; ChT, chemotherapy; DOC, died of other causes; DOD, died of disease; NED, no evidence of disease; NOS, not otherwise specified; RN, radical nephrectomy; RT, radiation therapy. Values are presented as numbers (%) unless otherwise indicated.

In general, osteosarcoma in the kidney grows aggressively and is extremely fatal, and the contiguous structures of the kidney, such as the spleen, liver, and adrenal gland, can easily be infiltrated. The prognosis of EOS in the kidney or other locations is poor, with approximately 86% of patients with EOS in the kidney having metastases (27). In our case, it was difficult to detect EOS because all biochemical blood test results were often normal, except serum alkaline phosphatase, which has a relatively poor diagnostic specificity. CT scans are considered to be relatively characteristic because most of the renal EOS presents as a mass in the kidney, which resembles a “sunburst” space-occupying lesion. Clinical manifestations, imaging such as CT and MR, and pathological examinations are necessary for the diagnosis of EOS. Allen et al. reported 26 patients with EOS and summarized the points of diagnosis for EOS. First, the mass appeared in soft tissue but did not adhere to the bone or periosteum; Second, pathological examination showed that the mass manifested as a sarcoma pattern; Third, the mass-produced a bone-like cartilage matrix (28). No definitive osseous matrix was observed in this case. Consequently, a multitude of diagnostic challenges have arisen in this particular instance.

Our patient underwent genetic testing after surgery. We analyzed 755 tumor-associated genes and identified three associated genes (*PIK3CA*, *CTCF*, and *RASA1*). *PIK3CA* has been reported in colorectal, ovarian, and breast cancers; a study by Ian G (29) showed that *PIK3CA* is an oncogene in ovarian cancer, greatly extending recent findings in breast cancer, and encodes a highly conserved zinc finger protein that has been implicated in diverse regular functions (30). Mutations in *RASA1* can cause capillary malformations, which may result from the loss of functional proteins produced by the genes (31). Since there was no clinical evidence to support the suspicion that she was sensitive to chemotherapy, the patient did not receive chemotherapy after surgery.

## Data availability statement

The raw data supporting the conclusions of this article will be made available by the authors, without undue reservation.

## Ethics statement

The studies involving humans were approved by The ethics committee of Zhuhai People’s Hospital. The studies were conducted in accordance with the local legislation and institutional requirements. The participants provided their written informed consent to participate in this study. Written informed consent was obtained from the participant/patient(s) for the publication of this case report.

## Author contributions

JD, GW, and JZ: manuscript writing. QZ: pathological review. JC, HL and RZ: manuscript revision. All authors contributed to the article and approved the submitted version.
